# Knockout of the KH-Type Splicing Regulatory Protein Drives Glomerulonephritis in MRL-Fas^lpr^ Mice

**DOI:** 10.3390/cells10113167

**Published:** 2021-11-14

**Authors:** Lisa Schmidtke, Myriam Meineck, Sabrina Saurin, Svenja Otten, Fabian Gather, Katharina Schrick, Rudolf Käfer, Wilfried Roth, Hartmut Kleinert, Julia Weinmann-Menke, Andrea Pautz

**Affiliations:** 1 Department of Pharmacology, University Medical Center, Johannes Gutenberg University Mainz, 55131 Mainz, Germany; lisa.schmidtke@web.de (L.S.); sabrinasaurin@web.de (S.S.); svotten@students.uni-mainz.de (S.O.); fabian.gather@anat.uni-freiburg.de (F.G.); katharinaschrick@gmail.com (K.S.); Rudi.kaefer@web.de (R.K.); kleinert@uni-mainz.de (H.K.); 2 First Medical Department, University Medical Center, Johannes Gutenberg-University Mainz, 55131 Mainz, Germany; Myriam.Meineck@unimedizin-mainz.de; 3 Institute of Pathology, University Medical Center, Johannes Gutenberg-University Mainz, 55131 Mainz, Germany; Wilfried.Roth@unimedizin-mainz.de

**Keywords:** systemic lupus erythematosus, glomerulonephritis, post-transcriptional regulation, cytokine, KSRP

## Abstract

KH-type splicing regulatory protein (KSRP) is an RNA-binding protein that promotes mRNA decay and thereby negatively regulates cytokine expression at the post-transcriptional level. Systemic lupus erythematosus (SLE) is an autoimmune disease characterized by dysregulated cytokine expression causing multiple organ manifestations; MRL-Fas^lpr^ mice are an established mouse model to study lupus disease pathogenesis. To investigate the impact of KSRP on lupus disease progression, we generated KSRP-deficient MRL-Fas^lpr^ mice (MRL-Fas^lpr^/KSRP^−/−^ mice). In line with the predicted role of KSRP as a negative regulator of cytokine expression, lupus nephritis was augmented in MRL-Fas^lpr^/KSRP^−/−^ mice. Increased infiltration of immune cells, especially of IFN-γ producing T cells and macrophages, driven by enhanced expression of T cell-attracting chemokines and adhesion molecules, seems to be responsible for worsened kidney morphology. Reduced expression of the anti-inflammatory interleukin-1 receptor antagonist may be another reason for severe inflammation. The increase of FoxP3^+^ T cells detected in the kidney seems unable to dampen the massive kidney inflammation. Interestingly, lymphadenopathy was reduced in MRL-Fas^lpr^/KSRP^−/−^ mice. Altogether, KSRP appears to have a complex role in immune regulation; however, it is clearly able to ameliorate lupus nephritis.

## 1. Introduction

Systemic lupus erythematosus (SLE) is a complex, systemic autoimmune disease that is characterized by the formation of autoantibodies (especially against nuclear antigens and double-stranded DNA), the appearance of immune complexes, and activation of the complement system in combination with inflammatory processes. Depending on the origin and location of the immune complex deposition, patients experience various organ manifestations (e.g., vasculitis, glomerulonephritis, arthritis, skin manifestations, etc.). Both the cells and structures of the non-specific immune defense (plasmacytoid and myeloid dendritic cells, macrophages, neutrophil granulocytes, toll-like receptors and complement system), as well as the specific immune defense (T and B cells), interact in the pathogenesis of SLE [1,2,3]. Glomerulonephritis, which occurs in approximately 50% of patients [4], is still a major risk factor for overall morbidity and mortality in SLE. Despite the recent advances in SLE therapy, a number of patients fail to respond, respond inadequately to the potent anti-inflammatory and immunosuppressive therapies, or have severe adverse effects [5,6]. To design new immunosuppressive drugs with improved safety profiles, it is necessary to extend our knowledge of lupus nephritis progression and systemic disease, which remains poorly understood [7].

MRL-Fas^lpr^ mice, a well-established lupus disease model, spontaneously develop a lupus-like syndrome very similar to human disease. Comparable to human lupus, circulating autoantibodies, complement activation, severe glomerulonephritis, splenomegaly, lymphadenopathy, skin lesions, and arthritis characterize the disease in MRL-Fas^lpr^ mice [8,9,10]. The dysregulated production of inflammatory cytokines contributes to immune dysfunction and organ damage in lupus disease and often correlates with disease activity and the severity of glomerulonephritis [3,11,12].

To avoid an overwhelming and destructive immune response, as seen in glomerulonephritis progression, tight regulation of cytokine expression by transcriptional-, post-transcriptional-, and post-translational mechanisms is necessary. Post-transcriptional regulation of cytokine expression often depends on RNA-binding proteins (RBP) [13]. These proteins either stabilize or destabilize cytokine mRNAs by binding to specific sequence elements, often AU-rich elements (ARE) located in the 3′-untranslated region (3′-UTR) of the mRNA.

KSRP (KH-type splicing regulatory protein, also named KHSRP or far upstream binding protein 2–FuBP2) is a multifunctional, single-stranded nucleic acid-binding protein involved in different post-transcriptional processes, such as the regulation of mRNA splicing, -stability and -translatability, and microRNA (miRNA) maturation. The protein binds to AREs are located in the 3′-UTR of cytokine mRNAs, such as TNF-α and type I interferons (IFN), and thereby recruit enzymes of the mRNA decay machinery [14,15]. Therefore, KSRP is an important negative regulator of pro-inflammatory gene expression. 

In contrast to its proposed role as an important negative regulator of pro-inflammatory gene expression [14], the knockout of the KSRP protein in C57BL/6 mice (KSRP^−/−^ mice) ameliorates rheumatoid arthritis progression [16,17]. In light of these unexpected results, we decided to further characterize the importance of KSRP for chronic inflammation. As SLE is an important chronic inflammatory autoimmune disease with a need to identify new therapeutic options, we generated MRL-Fas^lpr^/KSRP^−/−^ mice to study the consequences of KSRP knockout on lupus disease progression, especially with regards to glomerulonephritis. 

## 2. Materials and Methods

### 2.1. Materials

All oligonucleotides and dual-labeled probes used were purchased from Sigma, Deisenhofen, Germany. Complete EDTA-free protease and a phosphatase inhibitor cocktail were obtained from Roche Diagnostics, Mannheim, Germany. All cell culture grade plastic materials were obtained from Greiner, Solingen, Germany. The High-Capacity cDNA Reverse Transcription Kit was purchased from Applied Biosystems, Darmstadt, Germany. The M-MuLV reverse transcriptase and the Taq 2x Master Mix was obtained from New England Biolabs, Ipswich, USA. The AMPLIFYME SG Universal Mix was obtained from 7Bioscience, Neuenburg am Rhein, Germany.

### 2.2. Animal Experiments

To generate MRL-Fas^lpr^KSRP^−/−^ mice, we backcrossed the C57BL/6 KSRP^−/−^ mice [15] to the genetic background of MRL-Fas^lpr^ mice for the 10th generation (Jackson Laboratory, Stock No: 000485 Bar Harbor, ME, USA). The 10th generation on intercrosses with MRL-Fas^lpr^KSRP ^+/-^ animals was carried out in order to obtain homozygous MRL-Fas^lpr^KSRP ^+/+^ (wild-type, WT) and MRL-Fas^lpr^KSRP^−/−^ (knockout, KO) mice. The following oligonucleotides were used for genotyping the KSRP-locus: KSRP-wt-for GCGGGGAGAATGTGAAGG, KSRP-ko-for CTCCGCCTCCTCAGCTTG, and KSRP-wt/ko-rev GAGGCCCCTGGTTGAAGG. The following oligonucleotides were used for genotyping the FAS-locus: Fas-wt/ko-for GTAAATAATTGTGCTTCGT, Fas-wt-rev: TAGAAAGGTGCACGGG, and Fas-ko-rev CAAATCTAGGCATTAACA. 

All mice were housed in accordance with standard animal care requirements. The animal studies were approved 5th August 2014 and 10th July 2019 by the ethical board (3 177-07/G 14-1-054, G 19-1-002) and were performed in accordance with the German animal protection law and the guidelines for the use of experimental animals as stipulated by the Guide of Care and Use of Laboratory Animals of the National Institutes of Health.

### 2.3. Western Blot Experiments

Protein expression in mouse tissue was analyzed as described [18]. Total cell extracts were prepared using a RIPA detergent buffer (50 mM TRIS/HCl (pH 7.4), 150 mM NaCl, 1% NP-40, 2 mM EDTA, 10% Glycerin, complete protease inhibitor cocktail 1:50 and complete phosphatase inhibitor cocktail 1:10 (Roche Diagnostics, Mannheim, Germany). A polyclonal anti-GAPDH-antibody (14C10, Cell Signaling, Germany), monoclonal anti-GAPDH-antibody (SC-32233, Santa Cruz Biotechnology, TX, USA), a polyclonal KSRP-antibody (NBP-1-18910, Novus Biological, Germany) and a polyclonal mouse-IgG-antibody (A6782. Sigma-Aldrich, Germany) were used. Quantity One software (Bio-Rad, Munich, Germany) was used for quantification.

### 2.4. Examination of Lymphadenopathy

Lymphadenopathy was examined phenotypically over the lifespan from week 4 up to week 19. The size of the lymph nodes on the goiter, forelegs and hind legs was examined weekly at the same time by palpation and classified according to the severity of the swelling using a score from one to three: 0 = not tactual; 1 = Small and one-sided; 2 = moderate, on both sides; 3 = strong, on both sides. The examination was blinded. The values of the three areas examined were added, resulting in a maximum score of nine.

### 2.5. Renal Histopathology

Kidney pathology was assessed as previously described [19]. In brief, the kidneys were fixed in 10% neutral buffered formalin for 24 h and embedded in paraffin. Stained paraffin sections (4 μm) with periodic acid-Schiff reagent were used to assess glomerular pathology by scoring each glomerulus and periglomerular area on a semi-quantitative scale: 0 = normal [35 to 40 cells/glomerular cross section (GCS), no periglomerular infiltrates]; 1 = mild [glomeruli with few lesions showing slight proliferative changes, mild hypercellularity (41 to 50 cells/GCS), periglomerular infiltrates <5 cell layers]; 2 = moderate [glomeruli with moderate hypercellularity (51 to 60 cells/GCS, including segmental and/or diffuse proliferative changes, hyalinosis), periglomerular infiltrates 5–10 cell layers], 3 = severe [glomeruli with segmental or global sclerosis and/or severe hypercellularity ( >60 cells/GCS), necrosis, and crescent formation, periglomerular infiltrates >10 cell layer]. We scored 20 GCS/kidney. Interstitial/tubular pathology was assessed semi-quantitatively on a scale of 0 to 3 in 10 randomly selected high-power fields. We determined the largest and average number of infiltrates and damaged tubules and adjusted the grading system accordingly: 0 = normal, 1 = mild, 2 = moderate, 3 = severe. Perivascular cell accumulation was determined semi-quantitatively by scoring the number of cell layers surrounding most vessel walls (score: 0 = none, 1 = < 5 cell layers, 2 = 5 to 10 cell layers, 3 = > 10 cell layers). 

### 2.6. Immunostaining

Kidney tissue was processed and stained for the presence CD4 (clone L3T4) (553043, BD Pharmingen, San Diego, CA, USA), CD8a (clone 53-6.7) (553027 BD Pharmingen, San Diego, CA, USA), FoxP3 (clone FJK-16s) (145773-82, eBioscience, San Diego, CA, USA), F4/80 (clone Cl:A3-1) (MCA497G, Bio-Rad, formerly Serotec, Hercules, CA, USA), B220 (clone RA3-6B2) (553084, BD Pharmingen, San Diego, CA, USA), IFN-γ (clone XMG 1.2), (14-7311-81, eBioscience, San Diego, CA), and anti-mouse Ki-67 Ab (clone SP6) (RM-9106-S0, NeoMarkers by ThermoScientific), as described previously for the kidney [20]. Cryosections for CD4, CD8a, F4/80 and B220: tissue samples were embedded in O.C.T.-compound (4583, Tissue-Tek Sakura) and snap-frozen. Sections of 4 µm thickness were used for immunostaining. Paraffin sections for FoxP3, IFN-γ, Ki-67: Preparation as described above.

Immunofluorescence staining on renal cryosections was performed with IgG FITC antibodies. Renal cryo-sections (4 µm) were fixed with 20% Acetone/80% Methanol at −20 °C, blocked with 10% normal goat serum in 1 x PBS/1% BSA, and stained with anti-IgG DyLight® 488 antibody (abcam, ab96879) in the dilutions 1:50, 1:100, 1:200, 1:400, 1:800. After 1 h incubation at room temperature, the slices were counterstained and mounted with DAPI containing aqueous mounting media (H-1200, Vector Laboratories). IgG complex severity in glomeruli was scored: 0—none, 1—mild, 2—mediocre, 3—severe.

### 2.7. Analysis of Mrna Expression in Cells or Tissues of MRL-Fas^lpr^/KSRP^−/−^ or MRL-Fas^lpr^/KSRP^+/+^ Animals

To analyze the mRNA expression of different immune relevant genes in cells or tissues of MRL-Fas^lpr^/KSRP^−/−^ or MRL-Fas^lpr^/KSRP^+/+^ animals, we prepared total RNA by homogenizing the sample in a GIT-buffer [21] and isolated the RNA as described [22]. Gene expression in samples was quantified in a two-step real-time RT-PCR as previously described [23] with the oligonucleotides listed in Table 1. To calculate the relative mRNA expression, the 2^(−ΔΔC(T))^ method was used [24]. 

Specific mRNA expression was measured using the qRT-PCR method and normalized to RNA Polymerase II (Pol2A) or TATA Box-Binding Protein (TBP) mRNA expression.

### 2.8. FACS

Single-cell suspensions from kidneys were prepared and stained for intracellular and extracellular antigens as previously described [25]. In brief, capsules were removed, then kidneys were mashed through a 40 µm sieve and washed with PBS. Cells were collected by centrifugation. Red blood cells were lysed using an ACK lysing buffer (0.15 M Ammonium Chloride, 10 mM Potassiumhydrogencarbonate, 0.1 mM Na_2_EDTA, pH 7.4), and the remaining cells were washed in PBS. Cells were then stimulated with 1 µg/mL Ionomycin (Sigma) and 50 ng/mL PMA (Sigma) in RPMI/10% FBS and GolgiStop (BD Pharmigen) for 4 h at 37 °C in an incubator. FACS buffer (PBS, 5% FBS, 0.09% NaN_3_) was used to wash the cells. To detect Th1 (CD4^+^ IFNy^+^) and Tregs (CD4^+^ FoxP3^+^) subsets, the respective Phenotyping Kit by BD Pharmigen was used according to the manufacturer’s instructions. Th1: 10 µL Antibody cocktail was used per sample (Mouse Th1/Th2/Th17 Phenotyping Kit, 560758), Treg: 5 µL Antibody cocktail was used per sample (Mouse Th1/Treg Phenotyping Kit, 560767). 

### 2.9. Proteome Profiler^TM^ (R&D Systems)

To analyze pro-inflammatory protein expression in murine tissue samples, we used the Proteome Profiler^TM^ “Mouse Cytokine Array Panel A” according to the protocol of the manufacturer (R&D Systems, Wiesbaden, Germany). The Quantity One software 4.9.6 Basic (BioRad, Munich, Germany) was used for quantification.

### 2.10. Statistics

Data represent means ± SEM. Statistical differences were determined by the factorial analysis of variance followed by “Tukey’s” or “Dunnett’s” multiple comparison test. In the case of two means, classical *t*-test analyses were used. Two-way ANOVA analysis, followed by Bonferroni’s multiple comparisons test, was performed. All statistical analyses were performed using Graphpad Prism 7.0.

## 3. Results

**Figure 1:**
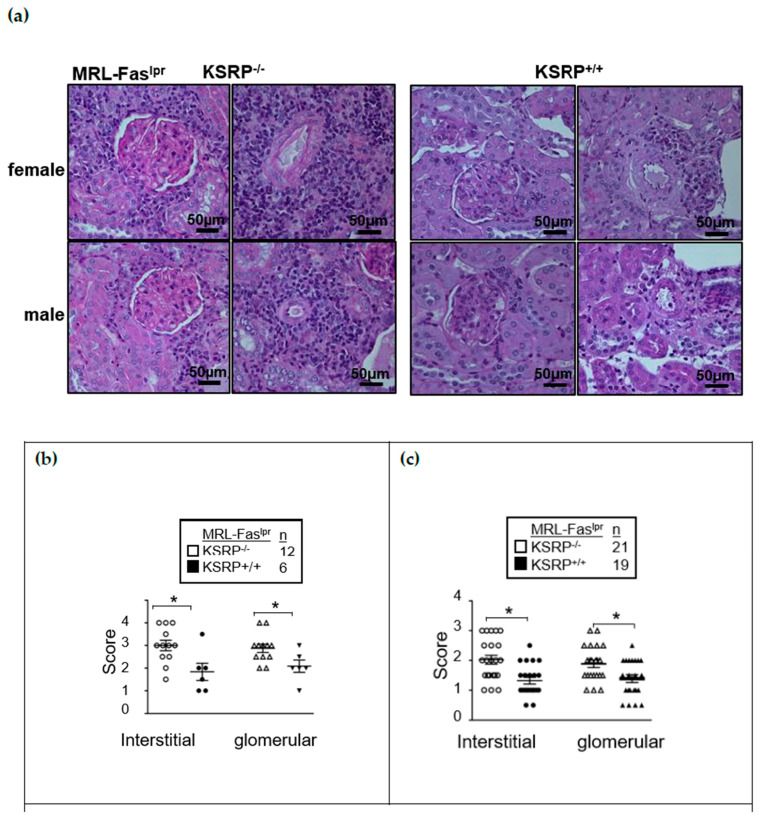
Kidney pathology in MRL-Fas^lpr^KSRP^−/−^ mice. PAS staining was used to assess glomerular pathology by scoring each glomerulus and periglomerular area on 19-week-old MRL-Fas^lpr^KSRP^+/+^ and MRL-Fas^lpr^KSRP^−/−^ mice, magnification 40×. Data presented are the mean ± SEM of the score with 6 to 12 females (**b**) and 19–21 male animals (**c**) per genotype (* *p* < 0.05 compared to WT, *t*-test).

To generate MRL-Fas^lpr^KSRP^−/−^ mice, C57BL/6-KSRP^−/−^ mice [15] were crossed to the genetic background of MRL-Fas^lpr^ mice. In the 10th generation, we started to analyze the phenotype of MRL-Fas^lpr^KSRP^−/−^ mice compared to MRL-Fas^lpr^ wild-type mice (MRL-Fas^lpr^KSRP^+/+^). The autoimmune symptoms in MRL-Fas^lpr^ mice develop gradually and become visible at the age of 3 months until they become severe at about 5 months. The knockout of the KSRP protein (Figure S1) enhanced glomerulonephritis severity in the kidneys of female and male MRL-Fas^lpr^KSRP^−/−^ mice at 19 weeks of age. Both in interstitial tissue as well as the glomeruli, we detected worsened kidney morphology via the evaluation of glomerular lesions, tubular damage, and the increased numbers of infiltrating cells in MRL-Fas^lpr^KSRP^−/−^ compared to MRL-Fas^lpr^KSRP^+/+^ mice, indicating more severe glomerulonephritis in MRL-Fas^lpr^KSRP^−/−^ mice. This was true for both sexes (Figure 1). Increased IgG deposition in the kidneys of female MRL-Fas^lpr^KSRP^−/−^ mice corroborate the more severe kidney phenotype (Figures S2 and S3). 

**Figure 2:**
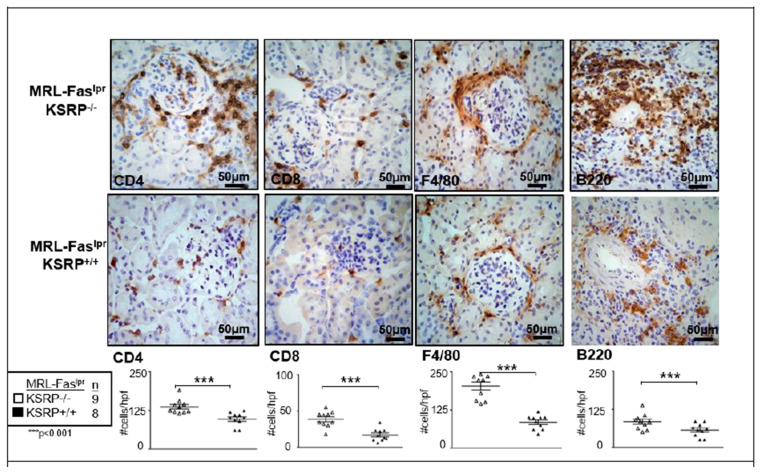
Frequencies of intra-renal CD4, CD8, F4/80 and B220 positive cells in the kidneys of 19-week-old female MRL-Fas^lpr^KSRP^−/−^ mice compared to MRL-Fas^lpr^KSRP^+/+^ mice Immunostaining was used to analyze intra-renal frequency of CD4, CD8, F4/80 and B220 positive cells in the kidneys of female 19-week-old MRL-Fas^lpr^KSRP^+/+^ and MRL-Fas^lpr^KSRP^−/−^ mice. Data presented are the mean + SEM of the score with 8 to 12 animals per genotype (*** *p* < 0.001 compared to WT, *t*-test). Representative image of immunostaining with 40× magnification.

To characterize the population of infiltrating cells in the kidneys of MRL-Fas^lpr^KSRP^−/−^ mice, we performed immunostaining, which revealed a significant increase of CD8^+^ T cells, F4/80^+^ macrophages, CD4^+^ T cells, and B220^+^ B cells in female MRL-Fas^lpr^KSRP^−/−^ compared to female MRL-Fas^lpr^KSRP^+/+^ mice (Figure 2). These results support our findings that the knockout of KSRP aggravates immune cell infiltration in the kidney of MRL-Fas^lpr^ mice. 

**Figure 3:**
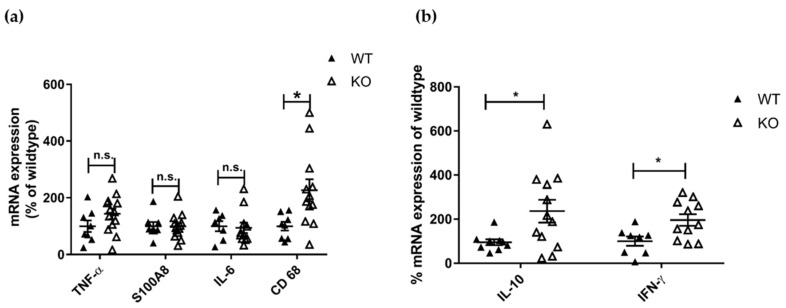
Analysis of mRNA expression of disease-relevant markers in the kidneys of 19-week-old female MRL-Fas^lpr^KSRP^−/−^ mice compared to MRL-Fas^lpr^KSRP^+/+^ mice. RNA was isolated from the kidneys (**a**,**b**) of 19-week-old female MRL-Fas^lpr^KSRP^−/−^ mice (KO) and MRL-Fas^lpr^KSRP^+/+^ mice (WT). The mRNA expression of TNF-α, S100A8, IL-6, CD68, IL-10 and IFN-γ was analyzed by qRT-PCR and normalized to the mRNA expression of Pol2a. Data presented are the mean ± SEM of the relative mRNA expressions based on WT (100%) with 8 to 12 animals per genotype (* *p* < 0.05; n.s.: not significantly different from WT, *t*-test).

We analyzed the mRNA expression of different lupus disease-related pro-inflammatory markers in the kidneys of MRL-Fas^lpr^KSRP^−/−^ and MRL-Fas^lpr^KSRP^+/+^ mice by qRT-PCR. The expression of TNF-α-, S100A8- and IL-6-mRNA was not affected by the knockout of the KSRP protein. In line with the immunostaining data, increased mRNA expression of the macrophage/monocyte marker CD68 was detected (Figure 3a). Furthermore, an approximately twofold increased expression of IL-10 and IFN-γ mRNA was detected in the kidneys of female MRL-Fas^lpr^KSRP^−/−^ mice (Figure 3b). 

**Figure 4:**
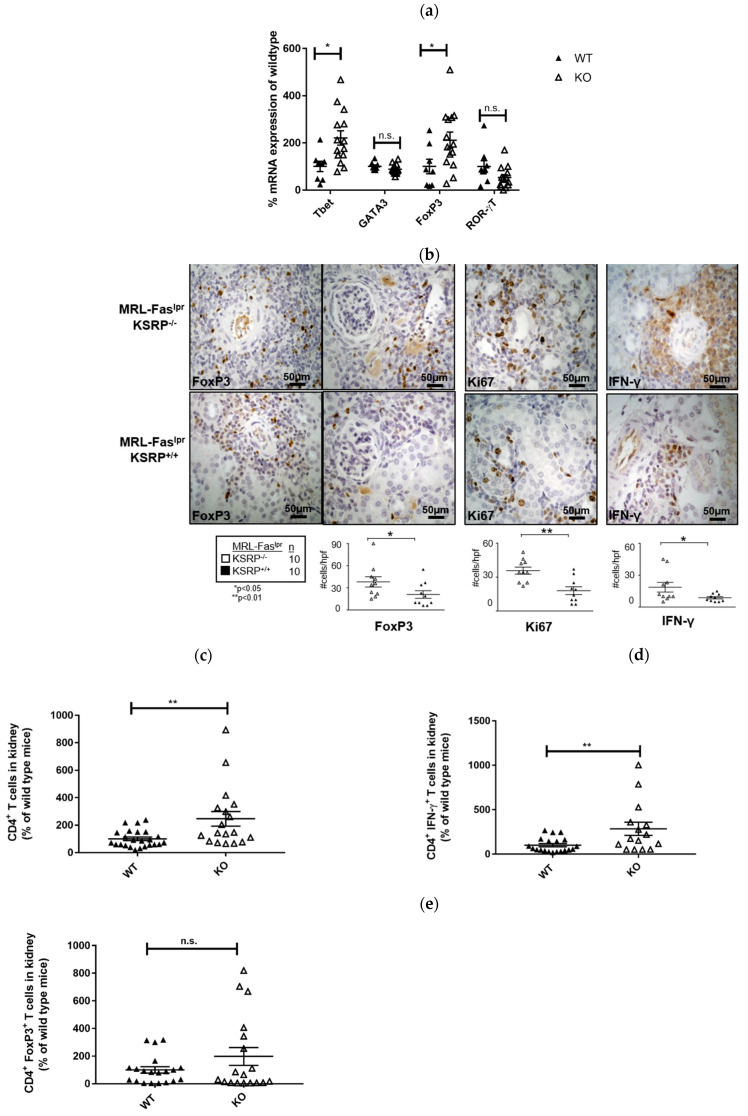
Characterization of T cell population in the kidneys of 19 and 15-week-old female MRL-Fas^lpr^KSRP^−/−^ mice compared to MRL-Fas^lpr^KSRP^+/+^ mice (**a**) RNA was isolated from the kidneys of 19-week-old female MRL-Fas^lpr^KSRP^−/−^ mice (KO) and MRL-Fas^lpr^KSRP^+/+^ mice (WT). T cell lineage-specific transcription factor mRNA expression of T-bet, GATA3, RORγT and FoxP3 were analyzed by qRT-PCR and normalized to the mRNA expression of Pol2a. Data presented are the mean ± SEM of the relative mRNA expressions based on WT female 19 weeks (100%) 9 to 14 animals per genotype (*: *p* < 0.05; n.s.: not significantly different from WT, *T*-TEST). (**b**) Immunostaining was used to analyze intra-renal frequency of FoxP3, Ki67 and IFN-γ producing cells in the kidneys of female 19-week-old MRL-Fas^pr^KSRP^+/+^ and MRL-Fas^lpr^KSRP^−/−^ mice. Data presented are the mean ± SEM of the score with 10 animals per genotype (**: *p* < 0.01, *: *p* < 0.05 compared to WT, TTEST). Representative image of immunostaining with 40x magnification. (**c**–**e**) FACS was used to analyze intra-renal frequency of CD4^+^, CD4^+^-IFN-γ^+^ and FoxP3^+^ cells in the kidneys of female 15-week-old MRL-Fas^lpr^KSRP^+/+^ and MRL-Fas^pr^KSRP^−/−^ mice. Data presented are the mean ± SEM of the relative cell numbers compared to MRL-Fas^lpr^KSRP^+/+^mice (19 to 23 animals per genotype) (**: *p* < 0.01, n.s.: not significant, compared to WT, *t*-test).

The immunostaining experiments presented in Figure 2 indicated increased numbers of T cells in the kidneys of 19-week-old MRL-Fas^lpr^KSRP^−/−^ mice. To characterize this T cell population, we analyzed the mRNA expression level of T helper (Th) cell lineage-specific transcription factors. Th1 cells require T-bet and Th2 cells GATA3 for their differentiation and activity. ROR-γT is important for Th17 cell development, whereas T regulatory (Treg) cells are often characterized by FoxP3 expression [26]. We detected increased T-bet and FoxP3 mRNA expression (increase about twofold) in the kidneys of MRL-Fas^lpr^KSRP^−/−^ mice, whereas expression of GATA3 and RORγT was not significantly altered (Figure 4a). This hints towards enhanced numbers of Th1 cells and Treg cells in the kidneys of MRL-Fas^lpr^KSRP^−/−^ mice. 

Immunostaining of intra-renal FoxP3 and IFN-γ expression in the kidneys of 19-week-old MRL-Fas^lpr^KSRP^−/−^ mice confirmed the mRNA expression analyses. In accordance, we detected an increased frequency of FoxP3 and IFN-γ positive stained cells compared to the control mice via FACS analysis (Figure 4b). 

Ki67 was used as a maker to determine proliferating cells. In the kidneys of MRL-Fas^lpr^KSRP^−/−^ mice, we detected an approximately twofold increase in Ki67 positive cells compared to MRL-Fas^lpr^KSRP^+/+^ mice (Figure 4b). This reflects the results of our PAS staining (Figure 1) and indicates that a larger number of active cells are present in the kidneys of MRL-Fas^lpr^KSRP^−/−^ mice. 

In 15-week-old MRL-Fas^lpr^KSRP^−/−^ mice, at an earlier time point of disease progression, we detected roughly two times more CD4^+^ T cells compared to the control mice in kidney cell homogenates by FACS analyses. Since the number of IFN-γ-producing CD4^+^ T cells was increased to a similar degree, these results indicate that the number of Th1 cells is increased in glomerulonephritis progression in MRL-Fas^lpr^KSRP^−/−^ mice. These data are in accordance with our mRNA expression analyses. The number of FoxP3 expressing CD4^+^ T cells was not significantly altered at this time point; however, a slight trend towards an increased frequency of these cells was also seen in 15-week-old MRL-Fas^lpr^KSRP^−/−^ mice (Figure 4c–e). 

**Figure 5:**
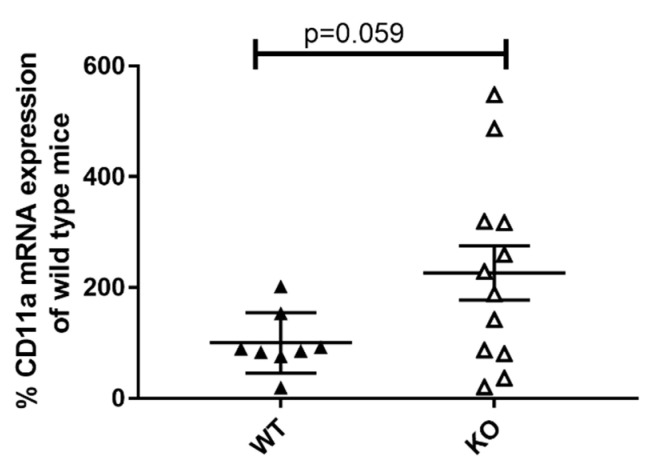
Characterization of CD11a expression in the kidneys of 19-week-old female MRL-Fas^lpr^KSRP^−/−^ mice compared to MRL-Fas^lpr^KSRP^+/+^ mice (a) RNA was isolated from the kidneys of 19-week-old female MRL-Fas^lpr^KSRP^−/−^ mice (KO) and MRL-Fas^lpr^KSRP^+/+^ mice (WT). CD11a mRNA expression was analyzed by qRT-PCR and normalized to the mRNA expression of Pol2a. Data presented are the mean ± SEM of the relative mRNA expressions based on WT females at 19 weeks (100%), 8 to 12 animals per genotype (*t*-test).

CD11a is a member of the β2 integrin family of adhesion molecules and is expressed on granulocytes, lymphocytes, and monocytes. It induces pro-inflammatory processes by promoting immune cell infiltration and cell–cell contact formation. Therefore, CD11a is essentially involved in immune cell activation [27]. Our results imply an increase in CD11a expression in the kidneys of 19-week-old female MRL-Fas^lpr^KSRP^−/−^ mice compared to MRL-Fas^lpr^KSRP^+/+^ mice (Figure 5). Similar results were obtained when analyzing the kidney tissue of six-week-old female MRL-Fas^lpr^KSRP^−/−^ mice and MRL-Fas^lpr^KSRP^+/+^ mice (data not shown). This KSRP-mediated difference could contribute to enhanced immune cell infiltration and activation in lupus-mediated nephritis. 

**Figure 6:**
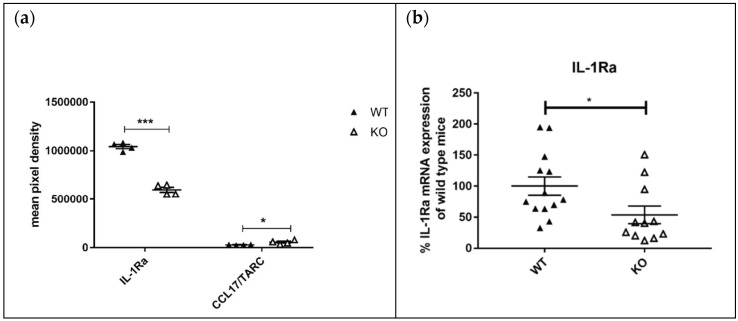
Analysis of chemokine and cytokine expression in the kidneys of MRL-Fas^lpr^KSRP^−/−^ mice (**a**) For the “mouse cytokine array” proteome profile analyses, proteins were isolated from the kidneys of MRL-Fas^lpr^KSRP^−/−^ mice (KO) compared to MRL-Fas^lpr^KSRP^+/+^ mice (three animals per group). The data show quantitative protein analyses of genotype-specific regulated cytokines and chemokines. All data (means ± SEM) show the absolute protein expression compared to MRL-Fas^lpr^KSRP^+/+^ mice (*** *p* < 0.001, *: *p* < 0.05 compared to WT, *t*-test, n = 3). (**b**) RNA was isolated from the kidneys of 19-week-old female MRL-Fas^lpr^KSRP^−/−^ mice (KO) and MRL-Fas^lpr^KSRP^+/+^ mice (WT). IL-1RA mRNA expression was analyzed by qRT-PCR and normalized to the mRNA expression of Tata-Box-Binding Protein (TBP). Data presented are the mean ± SEM of the relative mRNA expressions based on WT female 19 weeks (100%) 12 animals per genotype (*: *p* < 0.05; compared to WT 19 weeks, *t*-test).

Proteome profile analyses were performed to quantify the protein expression of some cytokines and chemokines. The most prominent result was that the interleukin-1 receptor antagonist (IL-1Ra) was one of the highest expressed proteins in the kidneys of MRL-Fas^lpr^ mice. In addition, IL1-Ra expression was down-regulated by 50% in MRL-Fas^lpr^KSRP^−/−^ mice compared to MRL-Fas^lpr^KSRP^+/+^ mice (Figure 6a). To validate our proteome profile analyses, we evaluated IL-1Ra mRNA expression in kidneys of MRL-Fas^lpr^KSRP^−/−^ and MRL-Fas^lpr^KSRP^+/+^ mice and also detected a two-fold down-regulation of IL-1 Ra as detected on protein level (Figure 6b). Moreover, we detected a twofold increase in the expression of CCL17, a chemokine that induces chemotaxis in T cells in MRL-Fas^lpr^KSRP^−/−^ mice (Figure 6a). 

**Figure 7:**
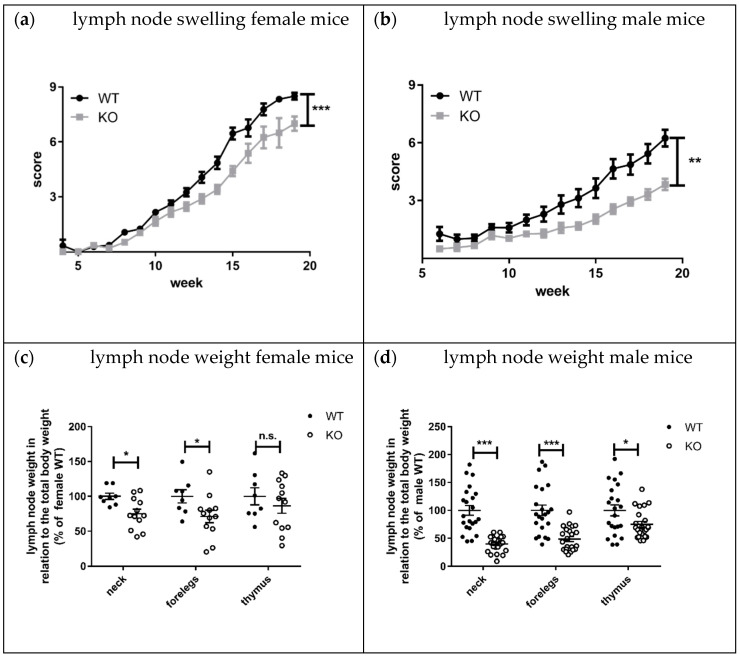
Lymph node swelling of female and male MRL-Fas^lpr^KSRP^−/−^ mice compared to the MRL-Fas^lpr^KSRP^+/+^ animals during the course of the disease. (**a**,**b**) Data presented are the mean values ± SEM of the lymph node swelling of MRL-FAS^lpr^KSRP^+/+^ (WT) and MRL-FAS^lpr^KSRP^−/−^ (KO). (**a**) 27‒30 females and (**b**) 25‒26 males (**: *p* < 0.01; ***: *p* < 0.001 compared to WT, two-way ANOVA). (**c**,**d**) The lymph nodes from the neck, front legs, and thymus of female and male 19-week-old MRL-FAS^lpr^KSRP^+/+^ and MRL-FAS^lpr^KSRP^−/−^ were weighed and set in relation to the total body weight. Shown are the mean values ± SEM of the relative organ weights in relation to the WT animals (100%) of 8 to 12 animals per genotype (**c**) and 19 to 21 per genotype (**d**) (*: *p* < 0.05; n.s.: not significantly different from WT, *t*-test).

**Figure 8:**
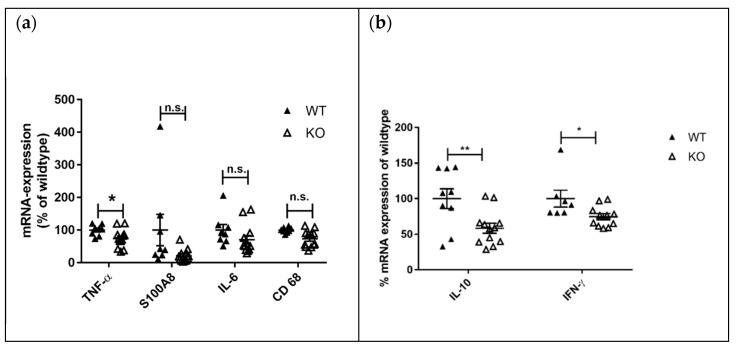
Analysis of mRNA expression of disease-relevant markers in the lymph nodes of 19-week-old female MRL-Fas^lpr^KSRP^−/−^ mice compared to MRL-Fas^lpr^KSRP^+/+^ mice. RNA was isolated from lymph nodes (**a**,**b**) of 19-week-old female MRL-Fas^lpr^KSRP^−/−^ mice (KO) and MRL-Fas^lpr^KSRP^+/+^ mice (WT). The mRNA expression of TNF-α, S100A8, IL-6, CD68, IL-10 and IFN-γ was analyzed by qRT-PCR and normalized to the mRNA expression of Pol2a. Data presented are the mean ± SEM of the relative mRNA expressions based on WT (100%) with 8 to 12 animals per genotype (**: *p* < 0.01; *: *p* < 0.05; n.s.: not significantly different from WT, *t*-test).

Lymphadenopathy, splenomegaly, and skin lesions are characteristic features of lupus disease in MRL-Fas^lpr^ mice [8]. Interestingly, the knockout of KSRP significantly reduced lymph node size and weight in both sexes compared to MRL- Fas^lpr^KSRP^+/+^ mice (Figure 7a–d). This effect seems to be more pronounced in male MRL- Fas^lpr^KSRP^−/−^ mice. The mRNA expression level of lupus disease-associated cytokines was analyzed in lymph nodes as well. We detected reduced mRNA expression of the inflammation markers TNF-α (*p* = 0.03) and S100A8 (*p* = 0.06) in MRL-Fas^lpr^KSRP^−/−^ compared to MRL-Fas^lpr^KSRP^+/+^ mice. IL-6 and CD68 expression were not altered (Figure 8a). Compared to the kidneys, we detected, in the lymph nodes, an opposite regulation of IL-10 and IFN-γ mRNA. Here, IL-10 and IFN-γ mRNA expression were down-regulated in the lymph nodes of MRL-Fas^lpr^KSRP^−/−^ compared to MRL-Fas^lpr^KSRP^+/+^ mice (Figure 8b). These results fit with our observation of reduced lymph node swelling in MRL-Fas^lpr^KSRP^−/−^ mice, indicating less severe inflammation in this tissue. Also, seizure of skin lesions in male MRL-Fas^lpr^KSRP^−/−^ mice was reduced compared to MRL-Fas^lpr^KSRP^+/+^ mice, whereas no, or even a slight, worsening of this symptom could be detected in female Fas^lpr^KSRP^−/−^ mice compared to MRL-Fas^lpr^KSRP^+/+^ mice (Figure S5). Other organ manifestations such as splenomegaly showed no difference between MRL-Fas^lpr^KSRP^−/−^ and MRL-Fas^lpr^KSRP^+/+^ mice (see Figure S4). Interestingly, on the one hand, we observed the improvement of lymphadenopathy and, on the other hand, the worsening of kidney pathology in MRL-Fas^lpr^KSRP^−/−^ mice. This indicates that the knockout of the KSRP protein has different effects on disease progression depending on the organ manifestation. 

## 4. Discussion

Innate and adaptive immune cells communicate via cytokines and chemokines, and therefore cytokine-mediated immunity is important in the pathogenesis of SLE [3]. Besides transcriptional regulation, the expression of these pro-inflammatory mediators is often controlled by modulation of their mRNA half-life. Therefore, proteins involved in the regulation of mRNA stability may modify immune cell function [28] and thereby may have an impact on SLE disease progression. 

KSRP has been described as an RBP important for the mRNA degradation of pro-inflammatory mediators [29]. Therefore, knockdown should enhance pro-inflammatory gene expression leading to more severe inflammation. In line with the accepted role of KSRP in the literature, we detected worsening symptoms of glomerulonephritis in MRL-Fas^lpr^KSRP^−/−^ mice indicated by enhanced immune cell infiltration, immune complex deposition, and increased IFN-γ production (Figure 1, Figure 2, Figure 3b, Figures S2 and S3). Numbers of CD4^+^ and CD8^+^ T cells, as well as macrophages, were increased, in the kidneys of MRL-Fas^lpr^KSRP^−/−^ compared to MRL-Fas^lpr^KSRP^+/+^ mice (Figure 2). Keep in mind that our control group, the 19-week-old MRL-Fas^lpr^ wild type mice with normal KSRP expression also developed severe glomerulonephritis accompanied by the increased infiltration of immune cells and expression of pro-inflammatory mediators compared to otherwise healthy mice. Loss of KSRP even increased this severe kidney inflammation by a further increase in immune cell infiltration and enhanced expression of pro-inflammatory mediators. Worsening of the existing glomerulonephritis in MRL-Fas^lpr^KSRP^−/−^ mice demonstrate the important role of KSRP in the regulation of inflammatory processes and immune cell function in vivo. The pre-existing pro-inflammatory phenotype in our control group may be one reason that the expression of some mediators analyzed in the kidneys, as well as IgG deposition, display no such huge differences between MRL-Fas^lpr^KSRP^+/+^ and MRL-Fas^lpr^KSRP^−/−^ mice (Figure 3, Figure S2). In addition, proteinuria was already on a high level in our 19-week-old female MRL-Fas^lpr^KSRP^+/+^ mice. Therefore, our semi-quantitative proteinuria measurement displayed no significant differences between 19-week-old female MRL-Fas^lpr^KSRP^−/−^ mice and this control group (data not shown). In accordance with our result of increased IFN-γ production in MRL-Fas^lpr^KSRP^−/−^ mice (Figure 3b), it has been demonstrated that in MRL-Fas^lpr^ mice, IFN-γ receptor signaling is important for lupus nephritis development [30] and increased IFN-γ expression in the kidneys, mainly driven by CD4^+^ T cells, has been described as well [31,32]. These results align with reports from lupus patients that high IFN-γ is associated with nephritis, arthritis, and lymphadenopathy [33]. 

A very recent report demonstrated that frequencies of nuclear antigen-specific CD4^+^ T cells correlated with human lupus disease severity. These autoreactive T cells produce effector cytokines such as IFN-γ or IL-10 [34]. This finding seems to be similar to the situation in MRL-Fas^lpr^KSRP^−/−^ mice. Besides an increased IFN-γ expression, we also detected elevated mRNA expression of IL-10 in the kidney of MRL-Fas^lpr^KSRP^−/−^ mice (Figure 3b). Similarly, we detected increased expression of FoxP3, a marker gene for Treg cells, and enhanced numbers of CD4^+^ Foxp3^+^ T cells in the kidney of MRL-Fas^lpr^KSRP^−/−^ compared to MRL- Fas^lpr^KSRP^+/+^ mice (Figure 4). These FoxP3^+^ cells might be the producers of IL-10. On the other hand, increased IL-10 might impair the function of those cells [35] and thus might explain the worsened kidney disease in MRL-Fas^lpr^KSRP^−/−^ mice. As we detected also increased numbers of B cells in the kidney of MRL-Fas^lpr^KSRP^−/−^ mice (Figure 2), this could also be the source of increased IL-10 production leading to enhanced autoantibody production responsible for worsening of kidney disease.

A number of reports describe that a dysbalance between T effector and Treg cells exist in autoimmune diseases such as rheumatoid arthritis or SLE that may result in impaired function of Tregs [36]. In the kidneys of MRL-Fas^lpr^KSRP^−/−^ mice, the ratio may be shifted towards Th1 effector cells, and therefore, the Treg cells present in the kidney of MRL-Fas^lpr^KSRP^−/−^ mice might be less effective to control or dampen lupus nephritis disease progression. To maintain this hypothesis, the suppressive effects of the Treg population of MRL-Fas^lpr^KSRP^−/−^ mice have to be evaluated. 

The expression of the inflammation marker S100A8 [19,37] was not increased in the kidneys of MRL-Fas^lpr^KSRP^−/−^ mice (Figure 3a). Instead, our analyses revealed increased expression of serum amyloid A3 (SAA3) (Figure S6). SAA proteins are acute-phase proteins, and an increased expression in MRL mice and humans correlates with greater severity of the disease [38,39]. In addition, it has been demonstrated that SAA3 expression is triggered by severe inflammation and most probably produced by macrophages [40], this correlates with the situation in the kidneys of the MRL-Fas^lpr^KSRP^−/−^ mice. It is known that SAA proteins are involved in the recruitment of immune cells to inflammatory sites [41]. Therefore, SAA3 could be one of the multiple factors that support the recruitment of immune cells to the kidneys in MRL-Fas^lpr^KSRP^−/−^ mice.

In our protein analyses, a clear reduction of IL-1Ra expression in MRL-Fas^lpr^KSRP^−/−^ mice was detectable. (Figure 6). Only a few reports about IL-1Ra in SLE exist. Some describe increased IL-1Ra levels in SLE patients. Other data do not provide evidence for a correlation between IL-1Ra levels and SLE disease activity [42]. One report speculates about a high level of IL-1Ra expression in MRL-Fas^lpr^ mice to downregulate IL-1–mediated inflammatory reactions [43]. In our proteome profile analyses, IL-1Ra was one of the highest expressed proteins in the kidney samples of MRL-Fas^lpr^ mice. Therefore, downregulation in KSRP knockout mice might reduce the putative protective effect of IL-1Ra. 

On the other hand, we detected significant upregulation of CCL17, also known as TARC, a chemokine that attracts T cells (e.g., Treg or Th2 cells) and is produced by dendritic cells, endothelial cells, or fibroblasts [44,45]. CCL17 has been studied predominantly in skin diseases, such as atopic dermatitis; however, elevated levels have also been reported in SLE patients. It might be possible that CCL17 is involved in the disease pathology of lupus nephritis by determining the extent of T cell migration or inflammation [46]. This hypothesis would fit with our results. Increased CCL17 levels in MRL-Fas^lpr^KSRP^−/−^ mice could explain the increase in Treg cell infiltration in those mice. In addition, in the lupus model of NZB/W F1 mice, increased CCL17 levels were detected [47]. 

Besides altered chemokine expression also changes in adhesion molecules could contribute to massive immune cell infiltration detected in the kidneys of the 19-week-old female MRL-Fas^lpr^KSRP^−/−^ mice. CD11a, a member of the β2 integrin family of adhesion molecules, regulates the recruitment of immune cells to sites of inflammation and promote cell–cell contact formation and thereby contribute essentially to immune cells, especially T cell activation. In the kidneys of 19-week-old female MRL- Fas^lpr^KSRP^−/−^ mice, CD11a mRNA expression seems to be increased (Figure 5), which could contribute to enhanced recruitment of lymphocytes and immune cell activation in the kidneys. In line with this result, it has been described that loss of CD11a protects MRL-Fas^lpr^ mice from autoimmune disease [48] and the other way round, CD11a overexpression can induce T cell auto-reactivity and B cell autoantibody production in lupus disease [49]. 

As previously mentioned, KSRP is also involved in miRNA maturation processes. The protein is a component of the Drosha and Dicer complex and promotes the maturation of miRNA precursors that contain a G-rich terminal loop. KSRP drives the maturation of pri-miRNAs into pre-miRNAs and of pre-miRNAs into miRNAs [50]. In the pathogenesis of lupus nephritis, altered levels of miRNAs have been described in murine models and human patients. This aberrant miRNA expression seems to modulate the immune cell function and inflammatory processes important for lupus nephritis disease progression [51]. There is an overlap of lupus disease-associated miRNAs and those whose maturation is regulated by KSRP (e.g., miR-155, miR183, miR148a, miR21, let-7 family, etc.). Therefore, it seems very likely that altered miRNA expression occurs in MRL-Fas^lpr^/KSRP^−/−^ animals and may contribute to the observed phenotype and changes detected in gene expression patterns. This point will be studied in detail in future experiments.

Lymphadenopathy was improved in MRL-Fas^lpr^KSRP^−/−^ compared to MRL-Fas^lpr^KSRP^+/+^ mice. The difference in lymph node size was striking, and molecular analyses clearly demonstrated the reduced expression of pro-inflammatory markers in lymph nodes of MRL-Fas^lpr^KSRP^−/−^ mice (Figure 7 and Figure 8). These results indicate that the immune-modulatory role of KSRP is quite complex and seems to be tissue-specific. Interestingly, in male mice, the reduced lymphadenopathy was even more pronounced than in female MRL-Fas^lpr^KSRP^−/−^ mice. These might imply that besides tissue, sex-specific effects of KSRP in lupus disease pathogenesis may exist.

Our initial screen of the new mouse strain MRL-Fas^lpr^KSRP^−/−^ identified cell types (CD4^+^ IFN-γ^+^ T cells, FoxP3^+^ T cells) and targets (IL-1Ra, CD11a) of interest for further studies. The data obtained so far will enable us to focus on specific cell types in the kidneys and lymph nodes that are involved in the development of the phenotype of the MRL-Fas^lpr^KSRP^−/−^ in the future. We believe that some of the results described above will even be more pronounced when they are analyzed in specific cell types isolated from kidneys or lymph nodes instead of analyzing them in whole tissue lysates. In addition, the study identified some molecules, such as IL-1Ra or SAA3, which have not yet been studied in lupus disease intensely; however, they seem to be important for lupus disease pathogenesis. Complex changes in gene expression patterns occur in MRL-Fas^lpr^KSRP^−/−^ mice. Therefore, the question is whether these are all direct KSRP-target genes or whether one or more upstream signaling events are modulated through the loss of KSRP. If KSRP regulates the expression of a “master cytokine”, such as IFN-γ, which is responsible for the polarization of an immune response (Th1 type), this might also explain the manifold changes in gene expression patterns detected in MRL-Fas^lpr^KSRP^−/−^ mice. 

## 5. Conclusions

In summary, our data indicate that KSRP has protective effects and ameliorate lupus nephritis by negative regulation of type II IFN expression in combination by hampering migration of immune cells, most probably by the modification of chemokine and adhesion molecule expression. The phenotype of the MRL-Fas^lpr^KSRP^−/−^ mice is interesting and demonstrates the first time that the post-transcriptional regulation of cytokine expression is an important field in lupus disease pathogenesis. Certainly, we have only just begun to investigate KSRP as a putative new therapeutic target for SLE treatment. Even in cases when KSRP cannot be directly therapeutically addressed, the new mouse model offers the possibility to identify other interesting target genes, which can be the starting point for the development of new alternative therapeutic pathways.

## Figures and Tables

**Table 1:** Oligos and probes used for qRT-PCR.

Oligo	5′-primer	3′primer	Probe
muCD11a	CCCTCCTAAGCGCAGATGAG	TGATCGCATGTCCGAGACAG	NA
muCD68	GGCAGCACAGTGGACATTC	CAATGATGAGAGGCAGCAAG	TCAGCTGCCTGACAAGGGACACTTG
muFOXP3	AGAAGCTGGGAGCTATGCAGG	GGGTTACGATGCAGCAAGAGC	CCTGGCTGGGAAGATGGCGCTG
muGATA-3	CTACCGGGTTCGGATGTAAGTC	GTTCACACACTCCCTGCCTTCT	AGGCCCAAGGCACGATCCAGC
muIFN-γ	TCAAGTGGCATAGATGTGGAAGAA	TGGCTCTGCAGGATTTTCATG	TCACCATCCTTTTGCCAGTTCCTCCAG
muIL-10	TGAAAATAAGAGCAAGGCAGTG	TCATTCATGGCCTTGTAGACAC	TGAGGCGCTGTCATCGATTTCTCCC
muIL-6	GAGGATACCACTCCCAACAGACC	AAGTGCATCATCGTTGTTCATACA	CAGAATTGCCATTGCACAACTCTTTTCTCA
mu-IL1Ra	GGAAAAGACCCTGCAAGATGC	TGGTCCTTGTAAGTACCCAGC	NA
muPol2a	ACCACGTCCAATGATATTGTGGAG	ATGTCATAGTGTCACACAGGAGCG	CTGGGCATTGAGGCTGTGCGGAA
muROR-γT	CACGGCCCTGGTTCTCAT	CAGATGTTCCACTCTCCTCTTCTCT	ATGCCAACCGTCCTGGGCTCC
muS100A8	CTCCGTCTTCAAGACATCGTTTG	TCATTCTTGTAGAGGGCATGGTG	CAATGCCGTCTGAACTGGAGAAGGCC
muSAA3	CCTGGGAGTTGACAGCCAAA	TCCGGGCAGCATCATAGTTC	NA
muT-bet	ACCAGAACGCAGAGATCACTCA	CAAAGTTCTCCCGGAATCCTT	CTGAAAATCGACAACAACCCCTTTGCC
muTBP	CTACCGTGAATCTTGGCTGT	CTCTTGGCTCCTGTGCACA	NA
muTNF-α	CATCCTTCTCAAAATTCGAGTGACAA	TGGGAGTAGACAAGGTACAACCC	CACGTCGTAGCAAACCACCAAGTGGA

## Data Availability

The data used to support the findings of this study are available from the corresponding author upon request.

## Supplementary Materials

The following are available online at https://www.mdpi.com/article/10.3390/cells10113167/s1, Figure S1: Confirmation of KSRP knockout in MRL-Fas^lpr^KSRP^−/−^ mice, Figure S2: IgG expression in kidneys of 19-week-old female MRL-Fas^lpr^KSRP^−/−^ mice, Figure S3: Immune complex deposition in the kidneys of 15-week-old female MRL-Fas^lpr^KSRP^−/−^ mice, Figure S4: Splenomegaly in 19-week-old MRL-Fas^lpr^KSRP^---^ mice, Figure S5: Skin lesion size in 19-week-old MRL-Fas^lpr^KSRP^−/−^ mice, Figure S6: Analysis of SAA3 mRNA expression in the kidneys of female 19-week-old MRL-Fas^lpr^KSRP^−/−^ mice compared to MRL-Fas^lpr^KSRP^+/+^ mice.
