# Supplementary material for: Knockout of the KH-Type Splicing Regulatory Protein Drives Glomerulonephritis in MRL-Faslpr Mice
Source: Cells. 2021 Nov 14;10(11):3167. doi: 10.3390/cells10113167 (PMC8624031; doi:10.3390/cells10113167)
Supplement: Supplementary file 1 [file cells-10-03167-s001.zip › cells-1440498-supplementary.pdf]

Supplemental Data

Supplemental Figure S1

Absence of KSRP protein in MRL-Fas^{lpr}/KSRP$^{-/-}$ was confirmed by western blot analyses using specific KSRP antibody.

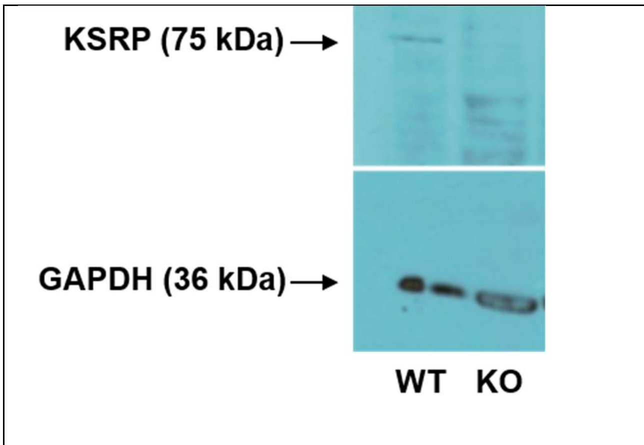

Suppl. Figure S1: Total protein was isolated from kidney of MRL-Fas^{lpr}/KSRP$^{+/+}$ (WT) and MRL-Fas^{lpr}/KSRP$^{-/-}$ (KO) mice as described in material and methods. Western Blot analyses was performed using a polyclonal KSRP antibody (NBP-1-18910, Novus Biologicals) and a polyclonal GAPDH antibody (14C10, Cell Signaling) to confirm equal protein load.

Supplemental Figure S2

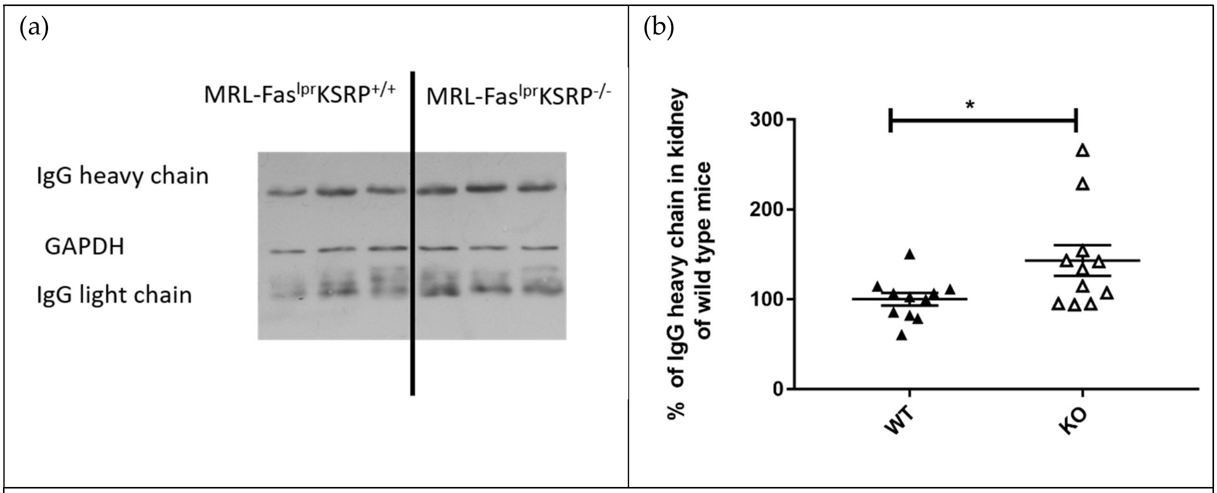

Suppl. Figure S2: Western Blot and Densitometry of IgG heavy chain content in the kidney of 19 week old female MRL-Fas^{lpr}KSRP$^{-/-}$ mice compared to the MRL-Fas^{lpr}KSRP$^{+/+}$ animals Total protein was isolated from kidney of 19-week-old female MRL-Fas^{lpr}/KSRP$^{+/+}$ (WT) and MRL-Fas^{lpr}/KSRP$^{-/-}$ (KO) mice as described in material and methods. (a) Western Blot analyses were performed using a polyclonal Mouse-IgG-antibody (A6782. Sigma-Aldrich, Germany) and a monoclonal anti-GAPDH-antibody (SC-32233, Santa Cruz Biotechnology, Texas, U.S.A.) to confirm equal protein load. Shown is one of three western blots investigated. (b) Densitometry analyses of the IgG heavy chain and GAPDH was performed using the QuantityOne software (Bio-Rad, Munich, Germany). Data presented are the mean values ± SEM of IgG heavy chain expression of 11 WT and 11 KO female mice (*: $p < 0.05$; from WT, t-test).

Supplemental Figure S3

(a)

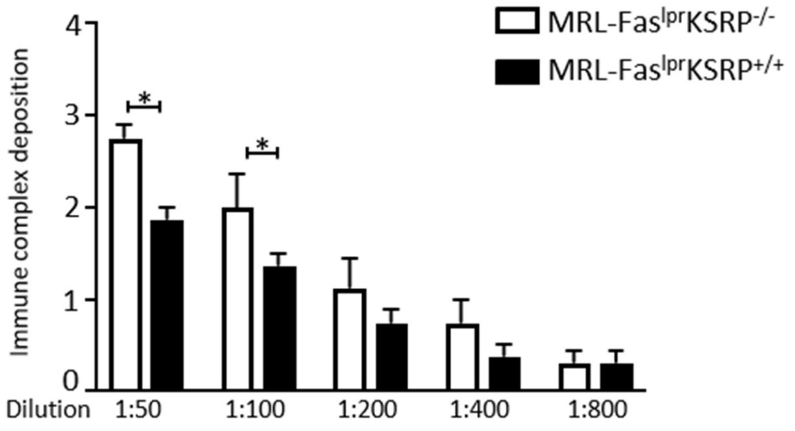

Suppl. Figure S3: Immune complex deposition in the kidney of 15 week old female MRL-Fas^{lpr}KSRP$^{-/-}$ mice compared to the MRL-Fas^{lpr}KSRP$^{+/+}$ animals. Renal cryo-sections (4 µm) were fixed with 20 % Acetone/80 % Methanol at -20 °C, blocked with 10 % normal goat serum in 1 x PBS/1 % BSA and stained with anti-IgG DyLight® 488 antibody (abcam, ab96879) in the dilutions 1:50, 1:100, 1:200, 1:400, 1:800. After 1 h incubation at room temperature, the slices were counterstained and mounted with DAPI containing aqueous mounting media (H-1200, Vector Laboratories). IgG complex severity in glomeruli were scored: 0 – none, 1 – mild, 2 – mediocre, 3 – severe. Data presented are the mean score + SEM of immune complex deposition of 4 female animals of each genotype (*: $p < 0.05$; from WT, Mann-Whitney-Test).

.

Supplemental Figure S4

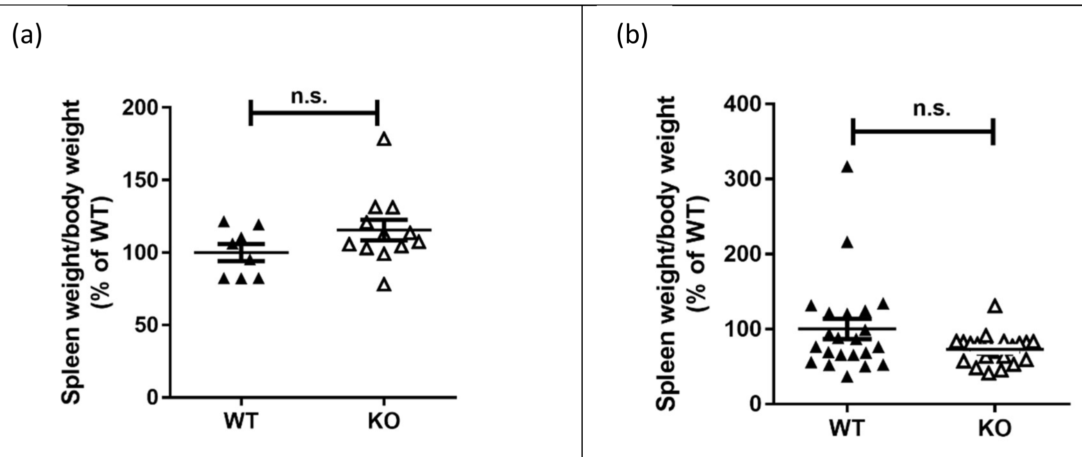

Suppl. Figure S4: Spleen weight in 19 week old female and male MRL-Fas^{lpr}KSRP$^{-/-}$ mice compared to the MRL-Fas^{lpr}KSRP$^{+/+}$ animals.

The spleen of female and male 19 week old MRL-FAS^{lpr}KSRP$^{+/+}$ and MRL-FAS^{lpr}KSRP$^{-/-}$ were weighed and the results were set in relation to the total body weight. Figure (a) and (b) show the mean values ± SEM of the relative organ weights in relation to the WT animals (100%) of 8 to 12 female animals (a) or 22-23 male animals (b) per genotype (n.s .: not significantly different from WT, t-test).

Supplemental Figure S5

(a) skin lesions female mice

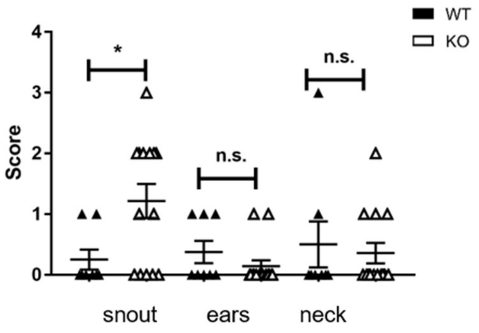

skin lesions male mice

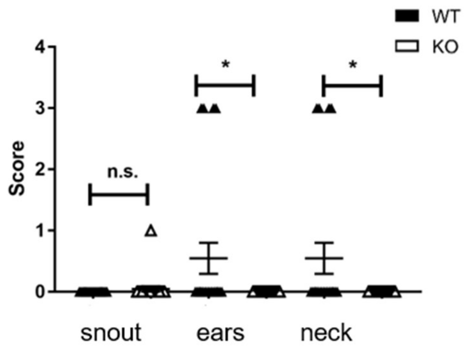

Suppl. Figure S5: Skin lesion size in 19 week old female and male MRL-Fas^{lpr}KSRP$^{-/-}$ mice compared to the MRL-Fas^{lpr}KSRP$^{+/+}$ animals MRL-Fas^{lpr} wild-type and KSRP-deficient mice were examined for seize of skin lesions at 19 weeks of age in a blinded manner. The skin lesions were evaluated with a score of 1 to 3. The values were summed up and the mean of all mice of the same sex and genotype was formed. Data presented are the mean values ± SEM of skin lesions of (a) 8-12 females and (b) 22-23 males (*: $p < 0.05$; n.s .: not significantly different from WT, t-test).

Supplemental Figure S6

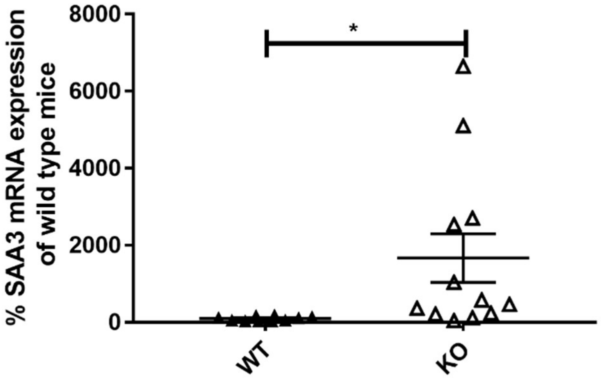

Suppl. Figure S6: Analysis of SAA3 mRNA expression in the kidney of female 19 weeks old MRL-Fas^{lpr}KSRP$^{-/-}$ mice compared to MRL-Fas^{lpr}KSRP$^{+/+}$ mice.

RNA was isolated from kidneys of 19 weeks old female MRL-Fas^{lpr}KSRP$^{-/-}$ mice (KO) and MRL-Fas^{lpr}KSRP$^{+/+}$ mice (WT). SAA3 mRNA expression was analyzed by qRT-PCR and normalized to the mRNA expression of TBP. Data presented are the mean ± SEM of the relative mRNA expressions based on WT female 19 weeks (100%) of 12 animals per genotype (*: $p < 0.05$ compared to WT 19 weeks, TTEST).

In global gene expression profiling experiments (data not shown) we detected increased expression of the serum amyloid protein A 3 (SAA3) in the kidney of MRL-Fas^{lpr}KSRP$^{-/-}$ mice compared to MRL-Fas^{lpr}KSRP$^{+/+}$ mice. This finding was confirmed in qRT-PCR analyses, were a fifteen-fold upregulation of SAA3 mRNA in the kidney of MRL-Fas^{lpr}KSRP$^{-/-}$ mice was detected (Supplemental Figure 6).
